# Maternal Exposure to Red Rooibos Does Not Alter Bone Development in Male or Female Sprague-Dawley Rat Offspring

**DOI:** 10.1016/j.cdnut.2023.100071

**Published:** 2023-03-30

**Authors:** Jenalyn L. Yumol, Michael D. McAlpine, Wendy E. Ward

**Affiliations:** 1 Department of Kinesiology, Brock University, St. Catharines, ON, Canada; 2 Department of Health Sciences, Brock University, St. Catharines, ON, Canada; 3 Centre for Bone and Muscle Health, Brock University, St. Catharines, ON, Canada

**Keywords:** bone structure, bone mineral density, microcomputed tomography, polyphenols, nutritional programming, red rooibos, tea

## Abstract

Maternal diet during pregnancy and/or throughout lactation provides a potential opportunity for nutritional programming of offspring bone development. Objectives of this study were to determine whether maternal consumption of red rooibos (RR) throughout pregnancy and lactation improved bone mineral density (BMD), bone structure, and bone strength in offspring and to determine potential sex-specific responses. Female Sprague-Dawley rats were randomly assigned to control water or RR in water (2600 mg/kg body weight/d) from prepregnancy through to the end of lactation. At weaning, offspring were fed AIN-93G diet until age 3 mo. Longitudinal assessment of the tibia demonstrated that maternal exposure to RR did not alter the trajectory of BMD or bone structure in male or female offspring compared with sex-specific controls at age 1, 2, or 3 mo or bone strength at age 3 mo. In conclusion, maternal exposure to RR did not program bone development in male or female offspring.

## Introduction

Many studies have shown that maternal diet can set a trajectory for bone health of offspring [1,2]. Findings from these human studies align with preclinical studies reporting effects of diet including the level and type of fat, low protein content, general undernutrition or overnutrition, as well as polyphenols, to program offspring bone health [[3], [4], [5], [6]]. Using the CD-1 mouse model, maternal and/or early-life exposure to polyphenols present in soy or citrus fruits has been shown to influence bone development through young adulthood, sometimes with sex-specific effects [5,6]. Maternal consumption of citrus flavonones resulted in divergent effects in male and female mouse offspring. From weaning to age 6 mo, offspring were fed AIN-93G control diet. Tibias from females who had been exposed to citrus flavonones through mother’s diet had lower bone mineral density (BMD) and trabecular number (Tb.N), less bone volume, and greater trabecular separation (Tb.Sp) at age 4 mo suggestive of a negative effect on bone development [5]. These differences were not present at 6 mo of age, demonstrating a transient effect. In contrast, male siblings had improved cortical structure of tibia compared with mice not exposed to flavonones [greater fraction of cortical bone (Ct.Ar/Tt.Ar) and cortical thickness (Ct.Th)] at age 2, 4, and 6 mo [6]. Moreover, at 6 mo of age, the trabecular structure of the femur neck and lumbar vertebra—sites that could not be measured in vivo—were higher than in control mice [6].

Flavonones have the potential for shared biological activity with other polyphenols found in teas that are derived from *Camellia sinensis* (white, yellow, green, oolong, black, and pu-erh tea) or *Aspalathus linearis* [green or red rooibos (RR) tea]. Extensive literature suggests positive effects of polyphenol interventions, with whole green tea or green tea extract, at attenuating loss of BMD and structure in ovariectomized rodent models [7,8]. Like ovariectomy, pregnancy and lactation also challenge the rodent skeleton. Using the Sprague-Dawley rat model, we previously studied if RR consumption supported recovery of mother’s BMD and bone structure after pregnancy and lactation [9]. In vivo microcomputed tomography was used to assess these outcomes before mating, at the end of lactation, and at monthly intervals until 4 mo after lactation (endpoint). Intervention with RR supported a partial recovery of BMD and trabecular bone structure at 2- and 3-mo after lactation. However, full recovery of these bone outcomes, as suggested by a level similar to the growth control, was not observed by the study endpoint [9]. The present study was an extension of this larger study in mothers, with a focus on offspring bone health. The objectives of this study were to determine whether maternal consumption of RR throughout pregnancy and lactation improved bone development and strength in male and female offspring at young adulthood, and to determine potential sex-specific responses. RR was selected because of the demonstrated stimulation of bone mineralization in vitro, possibly because of antioxidant activity [[9], [10], [11], [12]], and for its lack of caffeine content—an important consideration for fetal health.

## Methods

### Animals

This study was approved by the Animal Care Committee at Brock University, St. Catharines, Canada (Animal Use Protocol #18-03-02), and performed in accordance with the Canadian Council of Animal Care. Male and female Sprague-Dawley rats were the offspring of the mothers previously reported [9] and the study design is described here in brief: 5-week-old female Sprague-Dawley rats were acclimatized to the controlled environment for 1 week and then randomly assigned to receive 1 of 2 interventions (*n* = 14 rats/group): control water with 0 mg RR tea/kg body weight/d (CON) or water with 2600 mg RR tea/kg body weight/day. After 3 wk of receiving CON or RR, rats were mated and followed until 4 mo after lactation. These rats continued to receive CON or RR throughout the study, exposing their offspring to their assigned treatment in utero and during suckling. At weaning, 2 male and female offspring from each litter were selected based on having a body weight that was closest to the average body weight of the males or females, respectively, within a litter. Offspring siblings were housed in pairs, by sex, for socialization but only 1 rat per cage was randomly selected for analyses from weaning until age 3 mo. Offspring were fed AIN-93G diet (Envigo, Madison, WI) ad libitum and average body weight was recorded weekly.

### Longitudinal measurement of BMD, tissue mineral density, and structure of tibia analysis at 1, 2, and 3 mo of age

In a random order alternating between groups, longitudinal assessment of tibia BMD, tissue mineral density (TMD), and bone structure were conducted using in vivo microcomputed tomography (SkyScan 1176, Bruker microCT) [13], at 1, 2, and 3.25 mo of age (we were unable to scan at exactly 3 mo because of a scheduling challenge). Images were produced with an 18-μm resolution at a rotation step of 0.5 degrees over 360 degrees using 60 kV, 200 mA, and a 1-mm Al filter. Reconstruction included a smoothing correction of 2, ring artifact reduction of 8, beam hardening of 35, and dynamic image range of 0–0.0891168 (NRecon v.1.7.3.1.). Identical reorientation was conducted using Dataviewer (v.1.5.6.2, 64-bit) for quantification of BMD and trabecular bone structure (offset = 150 slices from the growth plate; height = 100 slices), as well as TMD and cortical bone structure at the proximal tibia (offset = 400 slices from the growth plate; height = 100 slices). CT Analyzer (v.1.17.7.2+, 64-bit) custom processing placed an adaptive thresholding of 37 (low) and 255 (high) and global thresholding of 56 (low) and 255 (high) for trabecular and cortical analysis, respectively.

### Trabecular structure of L4 and biomechanical strength of the femur at 3 mo of age

To accommodate the multiple days used for in vivo scans, euthanasia occurred at age 3.5 mo. Ex vivo analysis of L4 trabecular bone structure was conducted using high-resolution (9 μm) microcomputed tomography (SkyScan 1176, Bruker microCT). Scans were performed with a 1-mm Al filter and set to 68 kV and 368 uA for a 360-degree frame by a rotation step of 0.2 degrees. Images were reconstructed by a smoothing correction of 3, a ring artifact reduction of 8, beam hardening of 40, and a dynamic image range of 0–0.0712448. Trabecular bone structure of the vertebral body was quantified using adaptive thresholding: 57 (low) and 255 (high). Biomechanical bone strength at the femur midpoint was performed using a crosshead speed of 2 mm/sec (Materials Testing System, Model 4442, Instron Corp.) and quantified via associated Bluehill 2 software (Instron Corp.) [14].

### Statistics

The statistical power analysis (d = 1.64, alpha = 0.05, power = 0.80) was conducted a priori for a larger maternal study [9]. As previously described, one male and one female offspring from each litter were selected for analysis (*n*_CON_ = 13 offspring per sex because one mother did not become pregnant; *n*_RR_ = 14 males and *n*_RR_ = 13 females because one litter had only male offspring). A post hoc sensitivity analysis was performed for the intervention effect in offspring using G∗Power Version 3.1.9.7 [Cohen’s f = 0.51 (>0.4 for a large effect), alpha = 0.05, power = 0.95]. Results suggested that the sample size of 13–14 per group was appropriate to detect a large intervention effect. A 2 (CON, RR) × 2 (male, female) repeated measures linear model was used to determine whether there was a difference in tibia bone structure and BMD and body weight measures between male and female offspring of mothers exposed to CON or RR treatment at age 1, 2, or 3 mo. Significant differences were identified by a Bonferroni post hoc test. L4 bone structure outcomes and femur bone strength at 3 mo were assessed by a 2-way (treatment × sex) factorial analysis of variance. Analyses of the bone outcomes were performed using IBM SPSS Statistics 24 and statistical significance of *P* < 0.05. Data are presented as mean ± SEM.

## Results

Data described in the manuscript are publicly and freely available without restriction [18].

### Litter size and individual body weight

Mean litter size between maternal treatment groups did not differ [CON: 15 ± 2 (54% females); RR: 13 ± 2 (48% females); *P* > 0.05]. There was no treatment × age × sex interaction for body weight (*P* > 0.05) (Supplemental Figure). Main effects suggested that body weight significantly increased over time, and males were heavier than females from 1 mo onward (*P* < 0.05) (Supplemental Figure).

### In vivo tibia BMD, TMD, and bone structure

No significant treatment × age × sex interaction was observed for tibia bone structure outcomes (Table; Figure). There was no main effect of maternal exposure to RR on BMD, TMD, or bone structure but there was for age (*P* < 0.05) and sex (*P* < 0.05), with exception to Ct.Th (sex, *P* > 0.05). Regardless of sex, greater BMD and TMD, as well as improved bone structure [trabecular bone: greater bone volume fraction (BV/TV), trabecular thickness (Tb.Th), Tb.N and connectivity density and lower Tb.Sp; cortical bone: greater Ct.Ar/Tt.Ar, Ct.Th, periosteum perimeter (Ps.Pm), endocortical perimeter (Ec.Pm), and medullary area (Ma.Ar)] was demonstrated from age 1 to 3 mo because of growth. There were differences in bone development between sexes at age 2 and 3 mo; females had greater BMD, BV/TV, Tb.Th, and Tb.N and lower Tb.Sp, as well as greater TMD and Ct.Ar/Tt.Ar, but smaller Ma.Ar, Ps.Pm and Ec.Pm, when compared with males (*P* < 0.05).

**FIGURE:**
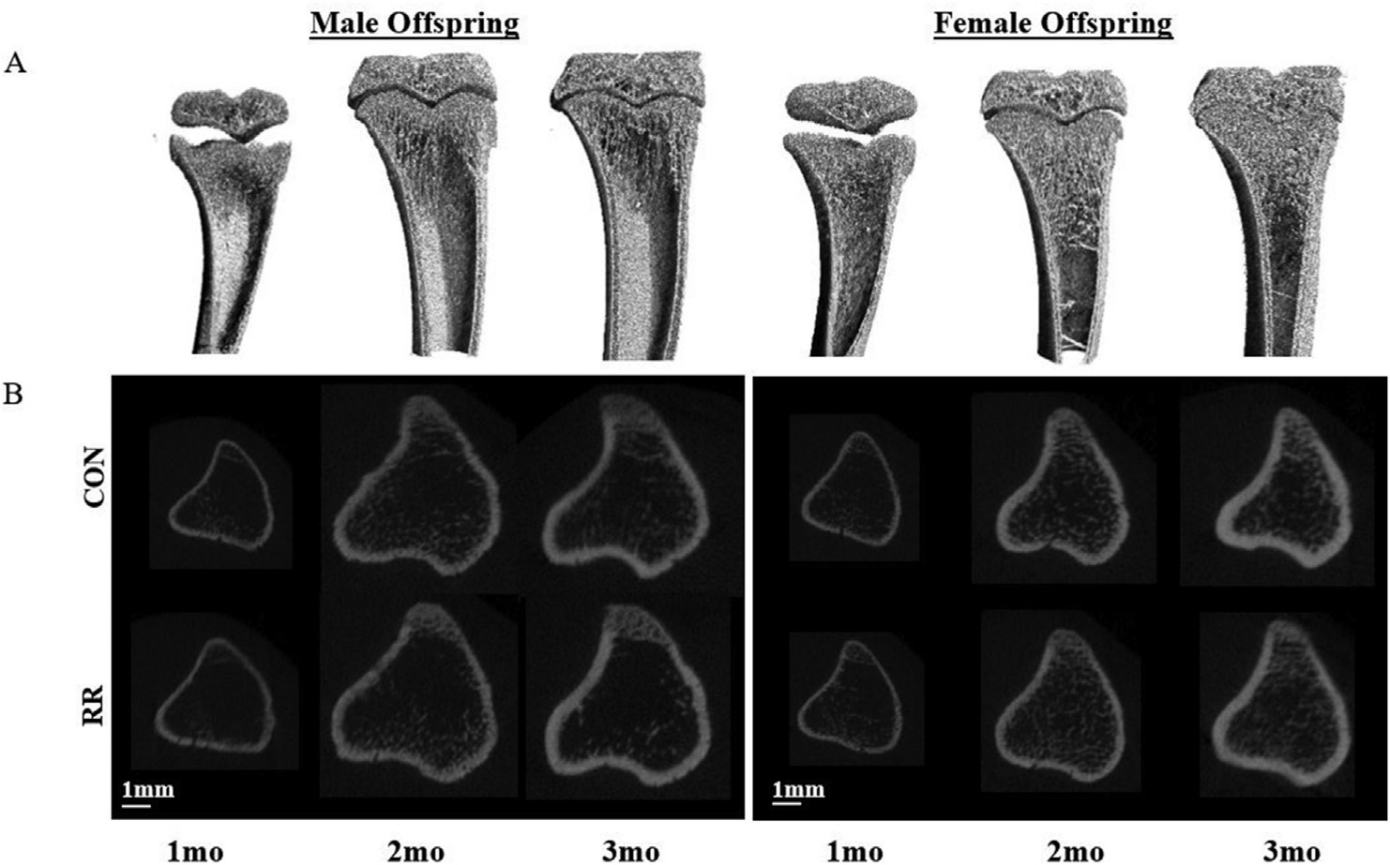
Representative images of the proximal tibia. (A) 3D images of the proximal tibia from selected male and female offspring from mothers who received CON (water) to show changes during development. (B) 2D images of the middle slice of the region of interest analyzed from offspring selected from mothers who received CON (water) or RR intervention. CON, control water; RR, red rooibos.

**TABLE:** Trabecular and cortical tibia bone structure outcomes at 1, 2, and 3 mo of age in male and female offspring from mothers who received CON (water) or RR intervention.

	Age (mo)	Maternal treatment groups							
		Male offspring		Female offspring		Repeated measures linear model, *P* values			
		CON	RR	CON	RR	Treatment	Age	Sex	Treatment × Age × Sex
		*n* = 13	*n* = 14	*n* = 13	*n* = 13				
Tibia (trabecular)^1^									
BMD, g/cm^3^	1	0.132 ± 0.003	0.127 ± 0.003	0.137 ± 0.003	0.131 ± 0.003	NS	0.000	0.000	NS
	2	0.187 ± 0.005	0.182 ± 0.006	0.248 ± 0.009	0.240 ± 0.012				
	3	0.188 ± 0.012	0.173 ± 0.008	0.312 ± 0.016	0.298 ± 0.014				
BV/TV, %	1	2.319 ± 0.148	2.153 ± 0.140	2.382 ± 0.190	2.152 ± 0.174	NS	0.000	0.000	NS
	2	6.032 ± 0.312	5.577 ± 0.302	8.630 ± 0.446	8.784 ± 0.649				
	3	6.518 ± 0.591	5.887 ± 0.468	12.121 ± 0.915	12.517 ± 0.725				
Tb.Th, mm	1	0.045 ± 0.0004	0.046 ± 0.0004	0.045 ± 0.001	0.044 ± 0.001	NS	0.000	0.002	NS
	2	0.052 ± 0.001	0.052 ± 0.0004	0.053 ± 0.0002	0.053 ± 0.001				
	3	0.053 ± 0.001	0.055 ± 0.001	0.057 ± 0.001	0.059 ± 0.001				
Tb.Sp, mm	1	0.874 ± 0.055	0.919 ± 0.045	0.775 ± 0.045	0.796 ± 0.041	NS	0.000	0.000	NS
	2	0.637 ± 0.040	0.670 ± 0.056	0.382 ± 0.039	0.386 ± 0.037				
	3	0.786 ± 0.081	0.864 ± 0.058	0.390 ± 0.060	0.351 ± 0.029				
Tb.N, mm^−1^	1	0.510 ± 0.031	0.469 ± 0.029	0.533 ± 0.041	0.484 ± 0.037	NS	0.000	0.000	NS
	2	1.167 ± 0.059	1.075 ± 0.059	1.633 ± 0.085	1.661 ± 0.110				
	3	1.231 ± 0.107	1.089 ± 0.100	2.129 ± 0.157	2.131 ± 0.119				
Tibia (cortical)^1^									
TMD, g/cm^3^	1	0.621 ± 0.004	0.610 ± 0.005	0.630 ± 0.007	0.613 ± 0.006	NS	0.000	0.000	NS
	2	0.716 ± 0.006	0.715 ± 0.010	0.812 ± 0.007	0.785 ± 0.007				
	3	0.830 ± 0.009	0.821 ± 0.014	0.954 ± 0.009	0.942 ± 0.009				
Ct.Ar/Tt.Ar, %	1	27.722 ± 1.078	27.179 ± 0.442	29.147 ± 1.047	28.049 ± 0.988	NS	0.000	0.000	NS
	2	34.232 ± 0.754	33.965 ± 0.972	38.618 ± 1.061	37.619 ± 1.051				
	3	41.414 ± 1.095	39.443 ± 0.697	50.211 ± 1.288	49.027 ± 0.873				
Ct.Th, mm	1	0.164 ± 0.004	0.161 ± 0.004	0.175 ± 0.005	0.163 ± 0.004	NS	0.000	NS	NS
	2	0.352 ± 0.006	0.352 ± 0.009	0.336 ± 0.007	0.337 ± 0.009				
	3	0.460 ± 0.011	0.447 ± 0.010	0.443 ± 0.009	0.439 ± 0.009				
Ps.Pm, mm	1	10.282 ± 0.197	11.240 ± 0.354	9.94 ± 0.349	10.366 ± 0.326	NS	0.000	0.000	NS
	2	16.756 ± 0.159	16.820 ± 0.285	14.328 ± 0.177	14.360 ± 0.203				
	3	18.547 ± 0.191	18.514 ± 0.271	14.431 ± 0.170	14.415 ± 0.228				
Ec.Pm, mm	1	8.671 ± 0.449	8.439 ± 0.387	7.720 ± 0.442	7.916 ± 0.470	NS	0.000	0.000	NS
	2	13.644 ± 0.217	13.869 ± 0.314	11.074 ± 0.265	11.217 ± 0.218				
	3	14.640 ± 0.290	14.655 ± 0.285	9.985 ± 0.262	10.090 ± 0.222				
Ma.Ar, mm^2^	1	4.198 ± 0.220	4.252 ± 0.156	3.766 ± 0.259	3.702 ± 0.198	NS	0.000	0.000	NS
	2	10.324 ± 0.328	10.554 ± 0.376	6.853 ± 0.323	7.183 ± 0.268				
	3	10.893 ± 0.458	11.605 ± 0.285	5.428 ± 0.299	5.622 ± 0.215				

BV/TV, percent bone volume; CON, control water; Ct.Ar/Tt.Ar, cortical area fraction; Ct.Th, cortical thickness; Ec.Pm, endocortical perimeter; Ma.Ar, medullary area; NS, not significant; Ps.Pm, periosteum perimeter; RR red rooibos; TMD, tissue mineral density; Tb.N, trabecular number; Tb.Sp, trabecular separation; Tb.Th, trabecular thickness.

1 Values are mean ± SEM (*P* > 0.05).

### Ex vivo L4 bone structure and peak load at femur midpoint

For L4, there was no maternal treatment × sex interaction or main effect for maternal treatment for BV/TV (CON_males_: 18.92 ± 0.85%; RR_males_: 19.07 ± 0.79%; CON_females_: 20.47 ± 0.73%; RR_females_: 20.86 ± 0.49%), Tb.Th (CON_males_: 0.08 ± 0.001 mm; RR_males_: 0.07 ± 0.001 mm; CON_females_: 0.07 ± 0.001 mm; RR_females_: 0.07 ± 0.001 mm), Tb.Sp (CON_males_: 0.32 ± 0.01 mm; RR_males_: 0.32 ± 0.009 mm; CON_females_: 0.28 ± 0.01 mm; RR_females_: 0.28 ± 0.004 mm), or Tb.N (CON_males_: 2.52 ± 0.10 mm^−1^; RR_males_: 2.57 ± 0.08 mm^−1^; CON_females_: 2.98 ± 0.08 mm^−1^; RR_females_: 3.03 ± 0.05 mm^−1^). Females had greater BV/TV, Tb.Th, and Tb.N and lower Tb.Sp, compared with males (*P* < 0.05). There were no differences in femur midpoint peak load because of RR (CON_males_: 177.58 ± 9.00 N; RR_males_: 178.75 ± 7.96 N; CON_females_: 136.52 ± 6.56 N; RR_females_: 146.56 ± 4.42 N, *P* > 0.05) though males demonstrated greater peak load at the femur midpoint (*P* < 0.05).

## Discussion

The consistent lack of effect in a comprehensive set of outcomes of bone health—including BMD, trabecular and cortical bone structure, and biomechanical bone strength—provides confidence that the early-life exposure to RR did not alter bone development in either male or female offspring. The lack of effect contrasts with our earlier studies with citrus flavonones and may be because of differences in species studied (mice versus rats) or the actual polyphenol profile and associated activities. Of note is that mothers who had or had not received the RR intervention throughout pregnancy and lactation had similar BMD and tibia bone structure at the time the pups were weaned [9]. Thus, if differences in outcomes of bone health in offspring were observed, these differences would likely not be the result of differences in maternal bone status, including mineral availability (mothers were not studied in the previous studies [5,6]). The lack of impact on bone outcomes of offspring from weaning until 3 mo of age may also be because of the healthy state of the pups and the control diet (AIN-93G). A dietary challenge may have provided an environment where the RR may have resulted in programming of bone development. A recent study of two maternal-offspring cohorts (Southampton Women’s Survey and Avon Longitudinal Study of Parents and Children) has shown a correlation between a higher late pregnancy dietary inflammatory index and offspring bone health at 9 years of age, specifically a lower bone mineral content and BMD [2]. In line with this study are previous findings that RR could benefit the bone through modulation of potential antioxidant activity. RR can stimulate in vitro osteoblast mineralization in a dose-dependent manner along with downregulation of osteopontin, increased alkaline phosphatase activity, and increased cell activity—possibly through higher antioxidant activity [9,11,12,15]. Osteoclast activity has also been shown to be inhibited by RR extract [16]. These findings suggest potential bone-promoting strategies beyond epigenetic mechanisms.

As expected, sex-specific differences in bone development were observed at age 2 and 3 mo but not at 1 mo of age. This is consistent with previous findings [17]. At age 3 mo, female rats had higher tibia BMD and more developed trabecular bone structure, whereas some cortical bone outcomes showed that male tibias were larger, and femurs were stronger at endpoint.

In conclusion, maternal exposure to RR throughout pregnancy and lactation does not benefit the trajectory of male or female offspring bone development through to age 3 mo. Moreover, this supplemental level of RR did not show adverse effects on bone development. Combining these findings with our earlier finding that maternal bone recovery was supported by RR consumption emphasizes the importance of studying the mothers and both male and female offspring to comprehensively assess the effect of polyphenols on bone health.

## Funding

This research was supported by a NSERC Discovery Grant to WEW.

## Author disclosures

The authors report no conflicts of interest.

## Data Availability

Data described in the manuscript are publicly and freely available without restriction at https://doi.org/10.5683/SP3/M7DW9V.

## References

[1] Masztalerz-Kozubek D., Zielinska-Pukos M.A., Hamulka J. (2021). Maternal diet, nutritional status, and birth-related factors influencing offspring’s bone mineral density: a narrative review of observational, cohort, and randomized controlled trials. Nutrients.

[2] Woolford S.J., D’Angelo S., Mancano G., Curtis E.M., Ashai S., Shivappa N. (2022). Associations between late pregnancy Dietary Inflammatory Index (DII) and offspring bone mass: a meta-analysis of the Southampton Women’s Survey (SWS) and the Avon Longitudinal Study of Parents and Children (ALSPAC). J. Bone Miner. Res..

[3] Mangu S.R., Patel K., Sukhdeo S.V., Savitha M.R., Sharan K. (2022). Maternal high-cholesterol diet negatively programs offspring bone development and downregulates hedgehog signaling in osteoblasts. J. Biol. Chem..

[4] Lanham S.A., Bertram C., Cooper C., Oreffo R.O.C. (2011). Animal models of maternal nutrition and altered offspring bone structure-bone development across the lifecourse. Eur. Cell. Mater..

[5] Sacco S.M., Saint C., LeBlanc P.J., Ward W.E. (2017). Maternal consumption of hesperidin and naringin flavanones exerts transient effects to tibia bone structure in female CD-1 offspring. Nutrients.

[6] Sacco S.M., Saint C., LeBlanc P.J., Ward W.E. (2018). Nutritional programming of bone structure in male offspring by maternal consumption of citrus flavanones. Calcif. Tissue Int..

[7] Shen C.-L., Smith B.J., Li J., Cao J.J., Song X., Newhardt M.F. (2019). Effect of long-term green tea polyphenol supplementation on bone architecture, turnover, and mechanical properties in middle-aged ovariectomized rats. Calcif. Tissue Int..

[8] Huang H.-T., Cheng T.-L., Lin S.-Y., Ho C.-J., Chyu J.Y., Yang R.S. (2020). Osteoprotective roles of green tea catechins. Antioxidants.

[9] McAlpine M.D., Yumol J.L., Ward W.E. (2021). Pregnancy and lactation in Sprague-Dawley rats result in permanent reductions of tibia trabecular bone mineral density and structure but consumption of red rooibos herbal tea supports the partial recovery. Front. Nutr..

[10] McAlpine M.D., Gittings W., MacNeil A.J., Ward W.E. (2019). Red rooibos tea stimulates osteoblast mineralization in a dose-dependent manner. Beverages.

[11] Nash L.A., Sullivan P.J., Peters S.J., Ward W.E. (2015). Rooibos flavonoids, orientin and luteolin, stimulate mineralization in human osteoblasts through the Wnt pathway. Mol. Nutr. Food Res..

[12] Nash L.A., Ward W.E. (2017). Tea and bone health: findings from human studies, potential mechanisms, and identification of knowledge gaps. Crit. Rev. Food Sci. Nutr..

[13] Longo A.B., Sacco S.M., Salmon P.L., Ward W.E. (2016). Longitudinal use of micro-computed tomography does not alter microarchitecture of the proximal tibia in sham or ovariectomized Sprague–Dawley rats. Calcif. Tissue Int..

[14] Sacco S.M., Jiang J.M.Y., Reza-López S., Ma D.W.L., Thompson L.U., Ward W.E. (2009). Flaxseed does not antagonize the effect of ultra-low-dose estrogen therapy on bone mineral density and biomechanical bone strength in ovariectomized rats. J. Toxicol. Environ. Health A.

[15] Nash L.A., Ward W.E. (2016). Comparison of black, green and rooibos tea on osteoblast activity. Food Funct.

[16] Moosa S., Kasonga A.E., Deepak V., Marais S., Magoshi I.B., Bester M.J. (2018). Rooibos tea extracts inhibit osteoclast formation and activity through the attenuation of NF-κB activity in RAW264.7 murine macrophages. Food Funct.

[17] de Bakker C.M.J., Zhao H., Tseng W.-J., Li Y., Altman-Singles A.R., Liu Y. (2018). Effects of reproduction on sexual dimorphisms in rat bone mechanics. J. Biomech..

[18] Yumol Jenalyn (2023).

