# Supplementary material for: Maternal Exposure to Red Rooibos Does Not Alter Bone Development in Male or Female Sprague-Dawley Rat Offspring
Source: Curr Dev Nutr. 2023 Mar 30;7(5):100071. doi: 10.1016/j.cdnut.2023.100071 (PMC10147958; doi:10.1016/j.cdnut.2023.100071)
Supplement: Multimedia component 1 [file mmc1.docx]

**Supplementary Figure 1.** Average body weight of male and female offspring from mothers that received CON (water) or RR from 4 to 14 weeks of age (study endpoint). Maternal intervention did not influence body weight of male or female offspring. Body weight increased over time and males were significantly heavier than females from age 1 month onward (p < 0.05). (CON) control water; (RR) red rooibos.
